# Predicting Poststroke Pneumonia in Patients With Anterior Large Vessel Occlusion: A Prospective, Population-Based Stroke Registry Analysis

**DOI:** 10.3389/fneur.2022.824450

**Published:** 2022-02-17

**Authors:** Martin A. Schaller-Paule, Christian Foerch, Ferdinand O. Bohmann, Sriramya Lapa, Björn Misselwitz, Konstantin Kohlhase, Felix Rosenow, Adam Strzelczyk, Laurent M. Willems

**Affiliations:** ^1^Department of Neurology, University Hospital Frankfurt, Goethe-University, Frankfurt am Main, Germany; ^2^Institute of Quality Assurance Hesse, Eschborn, Germany; ^3^Epilepsy Center Frankfurt Rhine-Main, University Hospital Frankfurt, Goethe-University, Frankfurt am Main, Germany

**Keywords:** infection, mechanical recanalization, endovascular thrombectomy, thrombolysis, neurocritical care, alteplase, complication

## Abstract

**Objective:**

To assess predictive factors for poststroke pneumonia (PSP) in patients with acute ischemic stroke (AIS) due to large vessel occlusion (LVO) of the anterior circulation, with special regard to the impact of intravenous thrombolysis (IVT) and endovascular treatment (EVT) on the risk of PSP. As a secondary goal, the validity of the A^2^DS^2^, PNEUMONIA, and ISAN scores in LVO will be determined.

**Methods:**

Analysis was based on consecutive data for the years 2017 to 2019 from the prospective inpatient stroke registry covering the entire federal state of Hesse, Germany, using the Kruskal-Wallis test and binary logistic regression.

**Results:**

Data from 4,281 patients with LVO were included in the analysis (54.8% female, median age = 78 years, range = 18–102), of whom 66.4% (*n* = 2,843) received recanalization therapy (RCT). In total, 19.4% (*n* = 832) of all LVO patients developed PSP. Development of PSP was associated with an increase in overall in-hospital mortality of 32.1% compared with LVO patients without PSP (16.4%; *p* < 0.001). Incidence of PSP was increased in 2132 patients with either EVT (*n* = 928; 25.9% PSP incidence) or combined EVT plus IVT (*n* = 1,204; 24.1%), compared with 2,149 patients with IVT alone (*n* = 711; 15.2%) or conservative treatment only (*n* = 1,438; 13.5%; *p* < 0.001). Multivariate analysis identified EVT (OR 1.5) and combined EVT plus IVT (OR 1.5) as significant independent risk factors for PSP. Furthermore, male sex (OR 1.9), age ≥ 65 years (OR 1.7), dysphagia (OR 3.2) as well as impaired consciousness at arrival (OR 1.7) and the comorbidities diabetes (OR 1.4) and atrial fibrillation (OR 1.3) were significantly associated risk factors (each *p* < 0.001). Minor stroke (NIHSS ≤ 4) was associated with a significant lower risk of PSP (OR 0.5). Performance of risk stratification scores varied between A^2^DS^2^ (96.1% sensitivity, 20.7% specificity), PNEUMONIA (78.2% sensitivity and 45.1% specificity) and ISAN score (98.0% sensitivity, 20.0% specificity).

**Conclusion:**

Nearly one in five stroke patients with LVO develops PSP during acute care. This risk of PSP is further increased if an EVT is performed. Other predictive factors are consistent with those previously described for all AIS patients. Available risk stratification scores proved to be sensitive tools in LVO patients but lack specificity.

## Introduction

Poststroke pneumonia (PSP) or stroke-associated pneumonia is a frequent and often preventable medical complication of acute ischemic stroke (AIS) that occurs in ~1 in 10 stroke patients and is among the major modifiable risk factors of in-hospital deaths related to stroke (1, 2). In addition to overall mortality, PSP considerably increases length of stay (LOS) and hospitalization costs, which underscores the need to screen for and prevent infections after stroke (3–5). Therefore, knowledge of predictive factors for PSP is a crucial prerequisite to identify high-risk patients and initiate prophylactic measures (6). As shown in the literature, risk factors commonly associated with PSP in AIS patients include advanced age, male sex, stroke severity as assessed by NIHSS, and clinical features such as atrial fibrillation, diabetes, and dysphagia (7, 8).

A subgroup with particular risk of PSP comprises AIS patients with an anterior large vessel occlusion (LVO); these patients commonly suffer more severe stroke syndromes and have higher overall mortality (9). Since 2015, in addition to intravenous thrombolysis (IVT), an increasing number of patients have benefitted greatly from endovascular treatment (EVT), which is primarily performed at high-volume stroke centers (10–12). However, EVT also bears potential health risks for patients due to the general anesthesia (GA) required for treatment and the possible need for prolonged invasive ventilation and intensive care, which was shown to predict unfavorable outcomes and increase pneumonia rates (13, 14). Hence, it is worthwhile to investigate which predictive factors for PSP apply to LVO patients and to what extent, and how application of recanalizing therapies (RCT) affects the overall risk for PSP in the individual patient. A previous retrospective analysis of 112 consecutive Chinese LVO patients who received EVT indicated that an unfavorable recanalization outcome is an independent risk factor for PSP (15). Analysis of a larger sample of LVO patients with and without RCT could provide more robust information regarding the predictive clinical factors of PSP.

Several risk stratification scores for PSP based on readily available clinical baseline data have been developed and repeatedly tested for AIS patients (7, 8). Well-validated tools include the A^2^DS^2^, PNEUMONIA, and ISAN scores, which are routinely used in the clinic to guide the monitoring of high-risk patients and inform decisions on prophylactic pneumonia management (16, 17). However, these tools were developed prior to the establishment of EVT in clinical practice and were not validated for the subgroup of stroke patients with LVO.

The primary goal of this study was to evaluate the predictive factors of PSP in stroke patients with LVO of the anterior circulation and to investigate the impact that RCT has on that risk. The secondary goal was to assess the validity of risk stratification scores in this subgroup of stroke patients.

## Methods

### Patient Cohort

This analysis was based on the 2017, 2018, and 2019 datasets from the mandatory prospective stroke inpatient quality assurance registry that included data for all patients with AIS with LVO of the anterior circulation treated at any of the 119 hospitals within the entire federal state of Hesse, Germany (6,285,000 inhabitants). Data entry into the registry is obligatory based on a federal contract on quality assurance in acute stroke care. Due to the anonymized data collection in the context of quality assurance measures, individual consent is not required. The data analysis was approved by the local ethics committee (313/16). All patients who met the following criteria were considered for the current evaluation: (1) discharge diagnosis of ischemic stroke (ICD-10: I63), (2) age ≥ 18 years, and (3) LVO in the anterior circulation [M1- or M2-occlusion of the middle cerebral artery (MCA) and carotid-T occlusion] confirmed by computed tomographic angiography (CTA), MR-angiography (MRA), or digital subtraction angiography (DSA). Information on presence of extracranial or intracranial carotid stenosis or occlusion was not available in the stroke registry, hence not considered in this study. The data included information regarding whether patients were transferred to another hospital for treatment. To avoid duplicate cases, all cases with notice of a transfer between hospitals were excluded. Stroke severity was assessed using the National Institutes of Health Stroke Scale (NIHSS, defined as mild ≤ 4, moderate 5–15, severe 16–20, very severe > 20) (18), and the level of disability was assessed using modified Rankin Scale (mRS). All patients that met inclusion criteria were analyzed in this study. Patients were not excluded in case of unfavorable outcome (e.g., palliative care) or event of death during hospital care (e.g., mRS = 6).

Diagnosis of PSP was entered manually into the database by stroke-unit physicians if criteria were met (see Section Poststroke Pneumonia Definition). Within the study centers, interventionalists were free to perform EVT under conscious sedation (CS) or GA and to individually choose the preferred method for EVT. Since neither type of anesthesia nor method of EVT were recorded in the registry, no further statement or analysis in this regard was feasible in the current study.

### Poststroke Pneumonia Definition

The presence of PSP is an obligatory data entry point in the quality assurance stroke registry Hesse. Clinical pneumonia was defined in the quality assurance registry Hesse documentation manual by using a combination of imaging, clinical, and laboratory criteria in accordance with guidelines from the Centers for Disease Control and Prevention (19), guidelines on hospital-acquired pneumonia from the German Society for Infectious Diseases (20), and guidelines from the American Thoracic Society (21). Poststroke pneumonia could only be coded if patients developed pneumonia during the hospital stay and did not fulfill the criteria for community-acquired pneumonia beforehand.

Clinically defined (poststroke) pneumonia could be coded if:

- Chest X-ray revealed either a new or progressive and persistent infiltrate, consolidation, cavitation, or pleural effusion

*and at least one of the following signs/symptoms occurred:*

- Fever (≥38.3°C or 100.94°F) without another explanation
- Leukopenia (<4,000 leukocytes/mm^3^) or leukocytosis (>12,000 leukocytes/mm^3^)
- For adults > 70 years of age: altered mental status not due to another cause (pneumonia-induced disorientation)

*with at least two of the following criteria:*

- New onset of purulent sputum or change in character of sputum
- New onset or worsening cough, dyspnea, or tachypnea
- Rales or bronchial breath sounds
- Worsening gas exchange [for example, O2 desaturations (e.g., PaO2/FiO2 ≤ 240), increased oxygen requirements, or increased ventilator demand]

In summary, documentation of pneumonia required a corresponding radiographic finding and a total of three of the additional criteria from the above list.

### Predictive Scores

#### A^2^DS^2^ Score

The A^2^DS^2^ score for the prediction of PSP was determined as presented in the literature (16), with a 10-point score including the items age ≥ 75 years (1 point), atrial fibrillation (1), dysphagia (2), and stroke severity as assessed by NIHSS (0–4 = 0, 5–15 = 3, ≥16 = 5 points). The cutoff point (A^2^DS^2^ ≥ 4) was chosen based on the original validation study of the score (16).

#### PNEUMONIA Score

The PNEUMONIA score was developed by Kwon et al. in 2006 and consists of five items, each of which is evaluated with a 0- or 1-point score, resulting in a minimum score of 0 points and a maximum score of 5 points. One point each is assigned for an NIHSS ≥ 11, age ≥ 65 years, male sex, need for mechanical ventilation, and dysphagia. In the initial study (17), all patients with a score of 5 developed pneumonia within the first 30 days after AIS; scores of 4, 3, 2, and 1 were associated with PSP in 74.2, 38.2, 5.3, and 0.9% of cases, respectively.

#### ISAN

The ISAN score was developed by Smith et al. in 2015 and uses four differently weighted items to calculate a sum score ranging from 0 to 21. Components of the score include age (<60 years = 0 points, 60–69 years = 3 points, 70–79 years = 4 points, 80–98 years = 6 points, >90 years = 8 points), sex (female 0 points, male 1 point), NIHSS at admission (0–4 = 0 points, 5–15 = 4 points, 16–20 = 8 points, >20 = 10 points), and mRS at admission [independent (mRS 0–1) = 0 points, not independent (mRS > 1) = 2 points] (22). The sum score allows classification of four different PSP risk groups, defined as low (ISAN = 0–5), medium (ISAN = 6–10), high (ISAN = 11–14), and very high (ISAN ≥ 15).

### Statistical and Graphical Analysis

Statistical analysis was performed with SPSS Statistics version 27 by the IBM Corporation (Armonk, NY, US) using the adequate non-parametric Kruskal-Wallis test for univariate analysis and binary logistic regression for multivariate analysis. Univariate analysis was used to identify predictive parameters at a *p*-value <0.05 that were included in the multivariate regression model. Since the multivariate analysis was based on values not corrected for multiple testing to address a possible suppressor effect, we refrained from additional *post-hoc* correction of the results of the univariate analysis that were used only descriptively (23). Regression coefficient (B) and odds ratio (Expβ) were used to interpret the significance of each factor with an OR of ≥1.25 and ≤ 0.75, respectively, that was considered clinically relevant (16). In addition, *p*-values <0.05 were considered statistically significant. The risk stratification scores were analyzed for their power to predict PSP in the study cohort, and receiver operating characteristic (ROC) curves were calculated. Sankey diagrams were created using SankeyMATIC by Steve Bogart (https://sankeymatic.com) and ROC curves were created using SPSS. Both figures were edited using Pixelmator Pro (Pixelmator Team, Vilnius, LTU).

## Results

### Patient Characteristics and Descriptive Statistics

**Figure 1:**
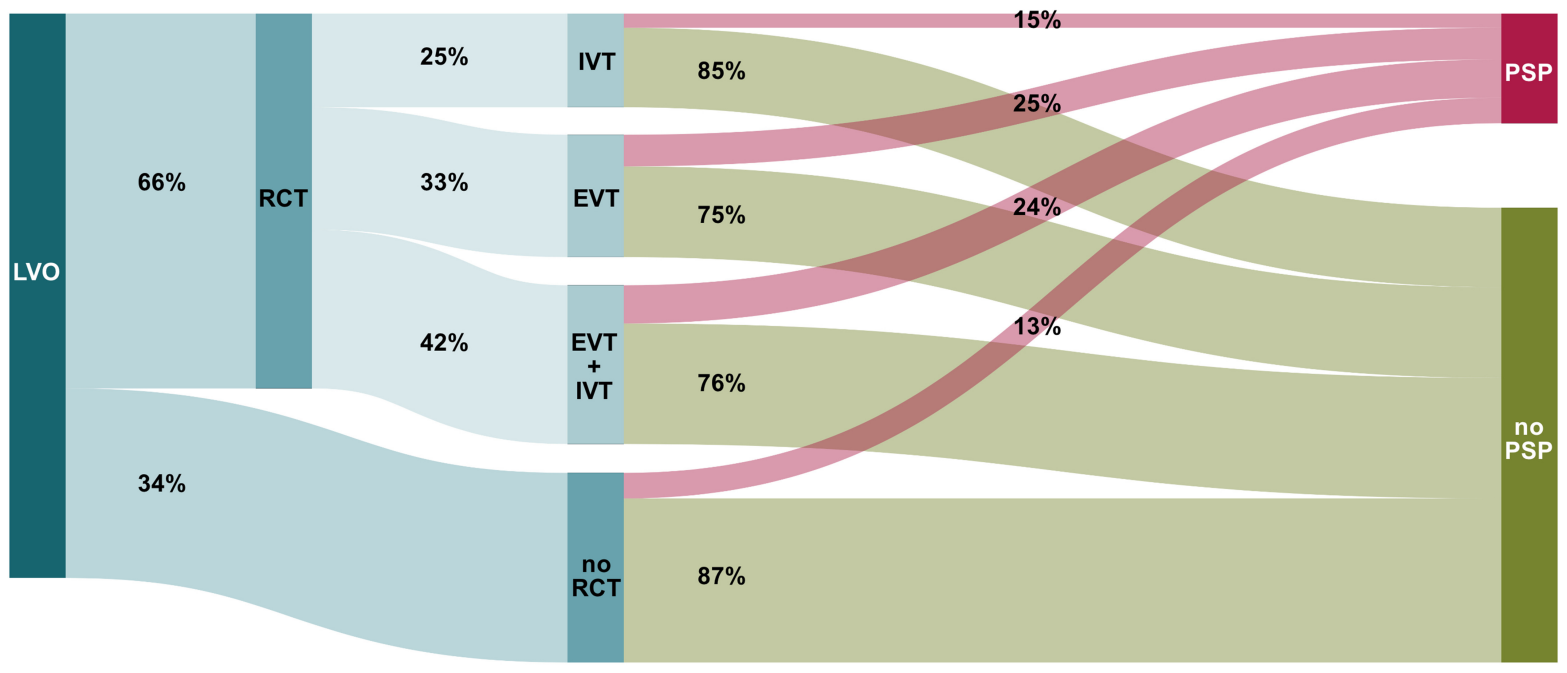
Sankey diagram illustrating the share of patients who do or do not develop poststroke pneumonia (PSP) in acute ischemic stroke (AIS) due to large vessel occlusion (LVO) of the anterior circulation, depending on the respective recanalizing therapy (RCT, recanalization therapy; IVT, intra-venous thrombolysis; EVT, endovascular treatment).

A total of 4,281 patients with AIS and LVO of the anterior circulation were included in the study analysis. Gender distribution showed 54.8% female and 45.2% male patients, with a mean age of 74.9 years (±13.3 years, median = 78 years, range = 18–102 years). Overall, 19.4% (*n* = 832) of patients developed PSP. Treatment with any RCT (IVT, EVT, or both) was reported in 66.4% of the patients (*n* = 2,843). In detail, 32.6% of patients received EVT (*n* = 928), 42.3% of patients received EVT plus IVT (*n* = 1,204), and 25.0% of patients received IVT (*n* = 711). Of the 1,438 patients treated conservatively, 13.5% developed PSP during their in-hospital stay. In comparison, 22.4% of patients with any RCT developed PSP during their in-hospital stay. PSP rates for patients receiving RCT were 25.9% for EVT, 24.1% for EVT plus IVT, and 15.2% for IVT. Comparison of *n* = 2132 patients undergoing EVT (either alone or with prior IVT) and patients without EVT (*n* = 2149) revealed significant differences regarding age, stroke severity, and need for intensive care treatment. A detailed overview of the two groups is provided in Supplementary Table 1. The distribution of patients regarding RCT and subsequent development of PSP is shown as a Sankey diagram in Figure 1.

### Univariate Analysis

Univariate analysis revealed sex, age category, and premorbid overall health condition as associated sociodemographic factors for PSP. Moreover, stroke severity (NIHSS) and presence of aphasia, dysphagia, dysarthria, motor impairment or impaired consciousness at admission were disease-specific factors associated with PSP. Regarding the LVO, the presence of MCA M1-occlusion was associated with a higher risk for PSP, whereas MCA M2-occlusion was accompanied by a lower risk for PSP. Atrial fibrillation, diabetes mellitus, and arterial hypertension were identified as comorbidities associated with PSP. Performance of RCT during the current admission, as well as EVT and EVT plus IVT, were associated with a higher incidence of PSP, whereas IVT alone was associated with a lower PSP prevalence. The results of the univariate analysis are displayed in Table 1.

**Table 1:** Univariate analysis of predictive factors for developing poststroke pneumonia after large vessel occlusion.

**Risk factor**		**Predictive of PSP**		**Not predictive of PSP**		***p*-value^a^**
		**%**	***n***	**%**	***n***	
**Sociodemographic factors**						
Sex	Female	17.2	404	82.8	1,941	**<0.001**
	Male	22.1	428	77.9	1,508	
Age, years	<65	12.6	110	87.4	764	**<0.001**
	≥65	21.2	722	78.8	2,685	
mRS at admission	≤ 3	8.1	84	91.9	953	**<0.001**
	≥4	23.1	748	76.9	2,496	
**Symptoms at admission**						
Initial NIHSS	≤ 4	6.2	39	93.8	587	**<0.001**
	5–15	16.2	320	83.8	1,659	
	16–20	28.2	313	71.8	795	
	≥21	28.3	160	71.7	406	
Aphasia	Yes	21.5	530	78.5	1,931	**<0.001**
	No	15.1	248	84.9	1,391	
Dysphagia	Yes	20.4	502	79.6	1,956	**<0.001**
	No	16.2	241	83.8	1,245	
Dysarthria	Yes	20.4	502	79.6	1,956	**0.001**
	No	16.2	241	83.8	1,245	
Motor impairment/paresis	Yes	20.5	772	79.5	2,988	**<0.001**
	No	7.8	34	92.2	400	
Impaired consciousness	Yes	29.9	328	70.1	769	**<0.001**
	No	15.8	504	84.2	2,680	
**Large vessel occlusion**						
ICA, carotid triangle	Yes	17.6	136	82.4	635	0.164
	No	19.8	696	80.2	2,814	
MCA, M1 segment	Yes	21.4	461	78.6	1,693	**0.001**
	No	17.4	371	82.6	1,756	
MCA, M2 segment	Yes	17.3	235	82.7	1,121	**0.018**
	No	20.4	597	79.6	2,328	
**Comorbidities**						
Atrial fibrillation	Yes	24.4	457	75.6	1,417	**<0.001**
	No	16.6	375	83.4	1,882	
Hypertension	Yes	20.7	716	79.3	2,739	**0.035**
	No	17.2	116	82.8	560	
Diabetes mellitus	Yes	24.8	232	75.2	704	**<0.001**
	No	18.8	600	81.2	2,595	
**Recanalization therapy**						
Recanalization therapy	Yes	22.4	638	77.6	2,205	**<0.001**
	No	13.5	194	86.5	1,244	
Only i.v. thrombolysis (IVT)	Yes	15.2	108	84.8	603	**0.002**
	No	20.3	724	79.7	2,846	
Only endovascular recanalization (EVT)	Yes	25.9	240	74.1	688	**<0.001**
	No	17.7	592	82.3	2761	
Failure of EVT (< TICI 2b)	Yes	35.8	57	64.2	102	**<0.001**
	No	18.8	775	81.2	3,347	
IVT and EVT	Yes	22.4	638	77.6	2,205	**<0.001**
	No	13.5	194	86.5	1,244	

*PSP, poststroke pneumonia; mRS, modified Rankin Scale; NIHSS, National Institutes of Health Stroke Scale; ICA, internal carotid artery; MCA, middle cerebral artery; IVT, intravenous thrombolysis; EVT, endovascular treatment; TICI, Thrombolysis in Cerebral Infarction scale*.

a *Calculated between both PSP categories using Kruskal-Wallis Test*.

*The level of significance of (P <0.05) are shown in bold*.

### Multivariate Analysis

Multivariate analysis resulted in a model that was statistically superior to the univariate analysis (*p* < 0.001, Cox & Snell R^2^ = 0.130, Nagelkerke's R^2^ = 0.195) and showed a small effect size (Cohen's D = 0.24). Within the model, male sex, age ≥ 65 years, presence of dysphagia at admission, impaired consciousness at admission, the comorbidities of atrial fibrillation and diabetes mellitus, and performance of RCT and EVT during the current admission contributed significantly to an increased risk for PSP, while a moderate stroke with NIHSS 5–15 was associated with a decreased risk for PSP. Notably, an EVT success rate worse than TICI 2b did not result in an increased risk for PSP. The results of the multivariate analysis are provided in Table 2.

**Table 2:** Multivariate analysis of possible predictive factors for developing poststroke pneumonia after large vessel occlusion.

**Parameter**	**B**	***p*-value^a^**	**OR**	**95% CI of Exp (B)**	
**Sociodemographic**					
Male sex	0.66	**<0.000**	1.94	1.60	2.34
mRS at admission ≥ 4	−0.23	0.169	0.79	0.57	1.10
Age ≥ 65 years	0.53	**0.002**	1.69	1.22	2.45
**Symptoms at admission**					
NIHSS ≤ 4 (minor)	−0.24	0.683	0.78	0.24	2.53
NIHSS 5–15 (moderate)	−0.63	**0.029**	0.53	0.30	0.94
NIHSS 16–20 (severe)	−0.27	0.069	0.76	0.57	1.02
NIHSS ≥ 21 (very severe)	0.07	0.638	1.07	0.81	1.42
Aphasia	−0.03	0.773	0.97	0.78	1.20
Dysarthria	−0.10	0.380	0.91	0.74	1.12
Dysphagia	1.17	**<0.000**	3.24	2.49	4.21
Paresis	0.05	0.859	1.05	0.62	1.78
Impaired consciousness	1.33	**0.003**	1.72	1.58	1.90
**Large vessel occlusion**					
MCA, M1	−0.15	0.275	0.87	0.67	1.12
MCA, M2	−0.01	0.970	1.00	0.75	1.32
**Comorbidities**					
Atrial fibrillation	0.27	**0.007**	1.31	1.07	1.59
Hypertension	−0.14	0.300	0.87	0.66	1.14
Diabetes mellitus	0.33	**0.002**	1.39	1.12	1.71
**Recanalization therapy**					
IVT or EVT	10.75	**0.000**	1.48	1.37	1.61
EVT	0.40	**0.009**	1.49	1.10	2.01
IVT	0.03	0.817	1.03	0.80	1.32
Failure of EVT	0.43	0.050	1.54	1.00	2.36

*PSP, poststroke pneumonia; mRS, modified Rankin Scale; OR, odds ratio; NIHSS, National Institutes of Health Stroke Scale; MCA, middle cerebral artery; IVT, intravenous thrombolysis; EVT, endovascular treatment; failure of EVT, outcome worse than TICI 2b*.

a *Calculated using a categorical binary logistic regression model*.

*The level of significance of (P <0.05) are shown in bold*.

### Mortality and Need for Intensive Care Treatment

The overall mortality of the cohort was 20.3% and was associated with prevalence of PSP (*p* < 0.001). In patients with PSP, mortality was increased at 32.1% when compared with a 16.4% mortality in patients without PSP during acute care. In addition, the need for intensive care was reported overall in 42.6% of LVO patients (*n* = 1761) and was significantly associated with an increased frequency (29.2%) of PSP (*p* < 0.001, Supplementary Table 1). After EVT, 65.7% of patients needed temporal intensive care treatment, compared with 67.3% after combined EVT plus IVT, 20.1% after IVT alone, and 17.5% with only conservative treatment (no RCT).

### Validation of Predictive Scores

#### A^2^DS^2^ Score

**Figure 2:**
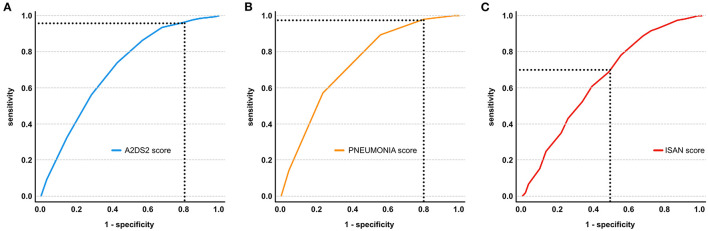
Receiver operating characteristic (ROC) curve illustrating the performance of A^2^DS^2^ **(A)**, PNEUMONIA **(B)**, and ISAN scores **(C)** in the prediction of PSP in AIS due to large vessel occlusion of the anterior circulation. The optimal sensitivity and 1 - specificity of the scores based on the ideal cutoff value derived from the ROC analysis are illustrated by the dashed lines.

For A^2^DS^2^ score, ROC analysis revealed an overall model quality of 0.68 with an AUC of 0.704 (95% CI 0.864–0.724). Based on the A^2^DS^2^ sum score of ≥4 as the cutoff value defined in the original study, a sensitivity of 96.1% and a specificity of 20.7% were calculated. See Figure 2A for an illustration of ROC curves.

#### PNEUMONIA Score

For PNEUMONIA score, ROC analysis showed an AUC of 0.734 (95% CI 0.715–0.753). Based on the analysis, a cutoff value of ≥2 showed a sensitivity of 78.2% and a specificity of 45.1%. See Figure 2B for an illustration of ROC curves.

#### ISAN Score

For ISAN score, ROC analysis showed an overall model quality of 0.63 with an AUC of 0.650 (95% CI 0.631–0.670). Based on the analysis, a cutoff value of ≥11 showed a sensitivity of 98.0% and a specificity of 20.0%. See Figure 2C for an illustration of ROC curves. Based on the ISAN risk categories, prevalence of PSP was 4.2% in the low-risk group, 13.0% in the medium-risk group, 22.3% in the high-risk group, and 29.0% in the very high-risk group.

## Discussion

PSP is a frequently reported complication in patients with AIS that has been associated with morbidity, mortality, increased LOS, and increased disease specific costs (3, 24, 25). In the literature, the reported incidence of PSP after AIS varies from 5.4 to 44% depending on the definition and clinical setting and is associated with various sociodemographic and disease-related aspects (1, 16, 24, 26, 27). In this study, 19.4% of LVO stroke patients developed PSP, which was significantly more frequent than the approximated 10% incidence of PSP following AIS of any etiology (1). Moreover, the data show a strong correlation between PSP occurrence and poor patient outcome. Presence of PSP was significantly associated with the increased necessity of ICU treatment and a two-fold increase in overall in-hospital mortality compared with AIS patients without PSP (32.1% mortality compared with 16.4% mortality), underlining the importance of identifying and quantifying risk factors for PSP development and implementing early detection mechanisms and preventive measures in clinical practice.

The multivariate analysis of LVO patients demonstrated that the baseline parameters of male sex, age above 65 years, dysphagia, and impaired consciousness at admission, as well as the comorbidities atrial fibrillation and diabetes, were significantly associated with PSP. These findings are largely consistent with previously reported predictive factors for PSP in all stroke patients (16, 17, 28). This indicates that LVO patients suffer more severe stroke syndromes and are at an overall higher risk of developing PSP, although the predictive risk factors remain mostly identical. In addition, multivariate analysis demonstrated that in LVO patients, the performance of EVT (OR 1.5) as well as the combination of EVT plus IVT (OR 1.5) were strong independent risk factors for PSP occurrence. While LVO patients who received conservative treatment only developed PSP in 13.5% of cases, application of EVT nearly doubled that risk to 25.9%. Notably, the group of patients that was selected for EVT differed in sociodemographic and clinical aspects (NIHSS, comorbidities), as well as in the affected vascular territories, from patients that did not receive EVT (Supplementary Table 1). However, these factors were part of the logistic regression that showed EVT as a strong independent risk factor. Though the design of this registry study does not allow us to prove causality, relevant factors that make EVT patients in particular more prone to developing PSP may be identified based on the literature.

In the presented cohort, an especially high percentage of patients (29.2%) developed PSP after receiving EVT and undergoing a consecutive ICU stay with artificial ventilation, which is in line with previous publications (24). A predominant factor for the necessity of ICU treatment—and development of PSP—is possibly the performance of general anesthesia (GA) with endotracheal intubation during EVT and the prolonged need for assisted ventilation. In comparison with EVT under conscious sedation (CS), GA was associated with lower rates of functional independence, higher incidence of PSP, and periprocedural hypotension (14). Especially prolonged weaning from the respirator may be a risk factor for PSP (13). In contrast, a comparative study stressed that the increased overall mortality of patients under GA relies on multiple factors and cannot be directly attributed to the higher rate of associated PSP, highlighting a need for further clinical studies (29).

In the univariate analysis (Table 1), failure of EVT—defined as a recanalization result worse than TICI 2b—was significantly associated with an increased risk of PSP. In the multivariate analysis of this study, however, failure of EVT was not associated with a significant additional risk of PSP. Though the item was indicative of an increased pneumonia risk in the multivariate regression model (OR 1.54; B 0.43), the effect did not reach a level of significance (*p* = 0.05). Thus, the recanalization result itself did not describe the risk of pneumonia as well as other items, such as male sex, age ≥ 65 years, dysphagia, impaired consciousness, or the fact that EVT and/or IVT was performed at all (Table 2). It can be assumed that the performance of EVT alone—especially in elderly patients with comorbidities—may contribute considerably to the risk of developing PSP, e.g., due to a decreased resilience and compensability of swallowing disorders (30, 31), and that these factors may outweigh the additional detrimental (or beneficial) effects of a good or poor recanalization result. In consequence, stroke physicians should be aware in their clinical routine that a patient undergoing EVT is always at an increased risk of developing PSP, even if the intervention is successful (32).

To prevent PSP, the administration of periprocedural antibiotics during EVT under GA has been discussed to some controversy. After three large phase 3 trials, a 2018 Cochrane meta-analysis showed no effect of preventive antibiotics against pneumonia in 4,488 acute stroke patients (33). However, the authors later stressed that a beneficial effect might still be applicable for certain subgroups of stroke patients and/or certain antibiotic regimens that have not yet been explored (34). In this regard, a *post-hoc* analysis of the PASS trial data revealed a beneficial effect of prophylactic intravenous ceftriaxone for patients after IVT (35). It is yet undetermined whether a subgroup of LVO patients with specific predictive risk factors undergoing EVT might also benefit from prophylactic intravenous antibiotics. A retrospective review of 549 cases of endovascular interventions (not exclusively stroke patients) with and without prophylactic antibiotics failed to show a significant beneficial effect (36). Therefore, a challenge for future studies is the identification of stroke patients that are most likely to benefit from prophylactic antibiotic treatment (34). For now, under consideration of the findings from the current study, stroke physicians should remain vigilant when treating at-risk patients with LVO and consider treatment with antibiotics at a low threshold upon signs of infection, if predictive risk factors for PSP apply.

Stroke patients show interconnected attributes that contribute to neurogenic dysphagia with (micro) aspiration and the likelihood of developing pneumonia, such as impaired consciousness, diminished airway reflexes, pharyngeal muscle relaxation, delayed swallowing reflexes, and volume depletion (29, 37). It is therefore important to determine which stroke patients are at an increased risk of developing PSP compared to others. Several risk stratification scores have been derived for the prediction of PSP in AIS patients and have been introduced into clinical practice. Their performance, however, was validated for all AIS patients and not tested for LVO stroke patients specifically. In this study, we aimed to assess the diagnostic utility of existing scores for LVO patients. Based on available data from 4,281 patients, the A^2^DS^2^, PNEUMONIA, and ISAN scores could be assessed, while other scores had to be dismissed due to insufficient information in our registry. The ROC analyses revealed considerable sensitivities of 96.1 and 98.2 for A^2^DS^2^ and ISAN scores, respectively, which were paired with low specificities of 20.7 and 20.0%, and in both cases were only close to acceptable model quality (0.68 and 0.63). In contrast, PNEUMONIA score showed an acceptable model quality (0.71) with a sensitivity of 78.2% and a specificity of 45.1%. In conclusion, all three score systems can be applied in clinical practice as screening tools for LVO stroke patients at risk of PSP, but their statistical limitations leading to false-positive (A^2^DS^2^ and ISAN) as well as false-negative (PNEUMONIA) results require careful interpretation.

Limitations of this study include the lack of information regarding the individual causality of PSP, as only frequencies could be correlated. The quality health care data was assessed state-wide in smaller hospitals and large stroke centers with possibly varying treatment options. Because of non-recorded clinical variables in the quality assurance stroke registry for Hesse, such as Glasgow Coma Scale at admission, systolic blood pressure in the first 24 h after admission, white blood cell count, blood glucose levels, stroke subtype, and history of pneumonia, the use of some PSP prediction scores, such as PANTHERIS, AIS-APS, or Chumbler's score, was not feasible in this study (26, 28, 38). Furthermore, no data was available on whether patients received GA or CS during EVT in the hospitals. It can only be assumed that a vast majority of patients receiving EVT were treated under GA because it is considered the standard procedure in Hesse, but no data on the modality was recorded. Noteworthy, presence of intracranial carotid occlusion could not be considered in this study, consequently no conclusion can be drawn for this subgroup of patients from this study.

A clear strength of this study lies in the predefined consensus on operational diagnostic criteria of pneumonia in the documentation manual. This contributes to a more homogeneous collective of PSP patients, which addresses a matter of criticism in prior registry studies on this topic (8, 27).

## Conclusion

Nearly one in five stroke patients with LVO developed PSP during acute care in this study. This risk of PSP was further increased if an EVT was performed, and if patients underwent ICU treatment. Other predictive factors were consistent with those previously described for all AIS patients, and the risk of developing PSP largely relied on the patients' predisposition and the severity of the stroke syndrome at admission. The risk stratification scores A^2^DS^2^, PNEUMONIA, and ISAN score proved to be sensitive tools in LVO patients but lacked specificity.

## Data Availability Statement

The data analyzed in this study is subject to the following licenses/restrictions: data will be made available after reasonable request due to German regulations on data protection. Requests to access these datasets should be directed to Martin A. Schaller-Paule, martin.schaller@kgu.de.

## Ethics Statement

The analysis was approved by the Institutional Review Board of the Ethical Committee at the University Hospital Frankfurt (313/16). Written informed consent for participation was not required for this study in accordance with the national legislation and the institutional requirements.

## Author Contributions

MS-P and LW conceived the study, performed the statistical analysis, and wrote the first draft of the manuscript. BM manages the state-wide prospective quality assurance database. AS gained ethical approval. FB and CF were involved in data preparation. MS-P, CF, FB, KK, BM, SL, FR, AS, and LW reviewed, revised, and edited the manuscript and approved the final version of the manuscript. All authors contributed to the article and approved the submitted version.

## Conflict of Interest

CF received speakers' honoraria from Boehringer Ingelheim and Bristol Myers Squibb and received honoraria for participating in advisory boards from Boehringer Ingelheim and Prediction Bioscience. FR reports personal fees from Angelini Pharma, Arvelle Therapeutics, Eisai GmbH, GW Pharmaceuticals companies, Medilearn India, Novartis, UCB Pharma, and research grants from the European Union, the German Research Foundation, the Federal state of Hesse and the Detlev-Wrobel-Fonds for Epilepsy Research Frankfurt. AS reports personal fees and grants from Angelini Pharma/Arvelle Therapeutics, Desitin Arzneimittel, Eisai, GW Pharmaceuticals companies, Marinus Pharma, UCB, UNEEG medical, and Zogenix. The remaining authors declare that the research was conducted in the absence of any commercial or financial relationships that could be construed as a potential conflict of interest.

## Publisher's Note

All claims expressed in this article are solely those of the authors and do not necessarily represent those of their affiliated organizations, or those of the publisher, the editors and the reviewers. Any product that may be evaluated in this article, or claim that may be made by its manufacturer, is not guaranteed or endorsed by the publisher.

## References

[1] Badve MS Zhou Z van de Beek D Anderson CS Hackett ML. Frequency of post-stroke pneumonia: systematic review and meta-analysis of observational studies. Int J Stroke. (2019) 14:125–36. 10.1177/1747493018806196 30346258

[2] Koennecke HC Belz W Berfelde D Endres M Fitzek S Hamilton F. Berlin stroke register, factors influencing in-hospital mortality and morbidity in patients treated on a stroke unit. Neurology. (2011) 77:965–72. 10.1212/WNL.0b013e31822dc795 21865573

[3] Finlayson O Kapral M Hall R Asllani E Selchen D Saposnik G. Stroke outcome research canada working, risk factors, inpatient care, and outcomes of pneumonia after ischemic stroke. Neurology. (2011) 77:1338–45. 10.1212/WNL.0b013e31823152b1 21940613

[4] Westendorp WF Nederkoorn PJ Vermeij JD Dijkgraaf MG van de Beek D. Post-stroke infection: a systematic review and meta-analysis. BMC Neurol. (2011) 11:110. 10.1186/1471-2377-11-110 21933425 PMC3185266

[5] Katzan IL Dawson NV Thomas CL Votruba ME Cebul RD. The cost of pneumonia after acute stroke. Neurology. (2007) 68:1938–43. 10.1212/01.wnl.0000263187.08969.45 17536051

[6] Katzan IL Cebul RD Husak SH Dawson NV Baker DW. The effect of pneumonia on mortality among patients hospitalized for acute stroke. Neurology. (2003) 60:620–5. 10.1212/01.WNL.0000046586.38284.60 12601102

[7] Zapata-Arriaza E Moniche F Blanca PG Bustamante A Escudero-Martinez I Ucles O. External validation of the ISAN, A2DS2, and AIS-APS scores for predicting stroke-associated pneumonia. J Stroke Cerebrovasc Dis. (2018) 27:673–76. 10.1016/j.jstrokecerebrovasdis.2017.09.059 29103860

[8] Kishore AK Vail A Bray BD Chamorro A Napoli MD Kalra L. Clinical risk scores for predicting stroke-associated pneumonia: a systematic review. Eur Stroke J. (2016) 1:76–84. 10.1177/2396987316651759 31008268 PMC6301233

[9] Malhotra K Gornbein J Saver JL. Ischemic strokes due to large-vessel occlusions contribute disproportionately to stroke-related dependence and death: a review. Front Neurol. (2017) 8:651. 10.3389/fneur.2017.00651 29250029 PMC5715197

[10] Saver JL Goyal M van der Lugt A Menon BK Majoie CB Dippel DW. Time to treatment with endovascular thrombectomy and outcomes from ischemic stroke: a meta-analysis. JAMA. (2016) 316:1279–88. 10.1001/jama.2016.13647 27673305

[11] Richter D Weber R Eyding J Bartig D Misselwitz B Grau A. Acute ischemic stroke care in Germany - further progress from 2016 to 2019. Neurol Res Pract. (2021) 3:14. 10.1186/s42466-021-00115-2 33789773 PMC8012074

[12] Weber R Bartig D Krogias C Richter D Hacke W Eyding J. Letter to the editor: analysis of stroke patient migration for mechanical thrombectomy and changes in neurointerventional center size in Germany. Neurol Res Pract. (2021) 3:32. 10.1186/s42466-021-00131-2 34092263 PMC8182903

[13] Fandler-Hofler S Heschl S Kneihsl M Arguelles-Delgado P Niederkorn K Pichler A. Ventilation time and prognosis after stroke thrombectomy: the shorter, the better! *Eur J Neurol*. (2020) 27:849–55. 10.1111/ene.14178 32065457 PMC7216995

[14] Eker OF Saver JL Goyal M Jahan R Levy EI Nogueira RG. Impact of anesthetic management on safety and outcomes following mechanical thrombectomy for ischemic stroke in SWIFT PRIME cohort. Front Neurol. (2018) 9:702. 10.3389/fneur.2018.00702 30210431 PMC6123376

[15] Zhu Y Gao J Lv Q Yin Q Yang D. Risk factors and outcomes of stroke-associated pneumonia in patients with stroke and acute large artery occlusion treated with endovascular thrombectomy. J Stroke Cerebrovasc Dis. (2020) 29:105223. 10.1016/j.jstrokecerebrovasdis.2020.105223 33066949

[16] Hoffmann S Malzahn U Harms H Koennecke HC Berger K Kalic M. Development of a clinical score (A2DS2) to predict pneumonia in acute ischemic stroke. Stroke. (2012) 43:2617–23. 10.1161/STROKEAHA.112.653055 22798325

[17] Kwon HM Jeong SW Lee SH Yoon BW. The pneumonia score: a simple grading scale for prediction of pneumonia after acute stroke. Am J Infect Control. (2006) 34:64–8. 10.1016/j.ajic.2005.06.011 16490608

[18] Ortiz GA Sacco RL. National institutes of health stroke scale (NIHSS). In: Wiley StatsRef: Statistics Reference Online. Balakrishnan N, Colton T, Everitt B, Piegorsch W, Ruggeri F, Teugels JL, editors. Hoboken, NJ: Wiley (2014).

[19] Anonymous Centers for Disease Control and Prevention: Pneumonia (Ventilator-associated [VAP] and non-ventilator-associated Pneumonia [PNEU]) Event Chapter 6: Pneumonia (PNEU) Event – January 2021. Atlanta, GA: Centers for Disease Control and Prevention, National Healthcare Safety Network (NHSN) (2021).

[20] Dalhoff K Abele-Horn M Andreas S Deja M Ewig S Gastmeier P. Update 2017 - S3 guideline of the German society for anaesthesiology and intensive care medicine, the German society for infectious diseases, the German society for hygiene and microbiology, the German respiratory society and the Paul-Ehrlich-society for chemotherapy, the German radiological society and the society for virology. Pneumologie. (2018) 72:15–63. 10.1055/s-0043-121734 29341032

[21] Kalil AC Metersky ML Klompas M Muscedere J Sweeney DA Palmer LB. Management of adults with hospital-acquired and ventilator-associated pneumonia: 2016 clinical practice guidelines by the infectious diseases society of America and the American thoracic society. Clin Infect Dis. (2016) 63:e61–e111. 10.1093/cid/ciw353 27418577 PMC4981759

[22] Smith CJ Bray BD Hoffman A Meisel A Heuschmann PU Wolfe CD. Can a novel clinical risk score improve pneumonia prediction in acute stroke care? A UK multicenter cohort study. J Am Heart Assoc. (2015) 4:e001307. 10.1161/JAHA.114.001307 25587017 PMC4330058

[23] Bursac Z Gauss CH Williams DK Hosmer DW. Purposeful selection of variables in logistic regression. Source Code Biol Med. (2008) 3:17. 10.1186/1751-0473-3-17 19087314 PMC2633005

[24] Hannawi Y Hannawi B Rao CP Suarez JI Bershad EM. Stroke-associated pneumonia: major advances and obstacles. Cerebrovasc Dis. (2013) 35:430–43. 10.1159/000350199 23735757

[25] Wilson RD. Mortality and cost of pneumonia after stroke for different risk groups. J Stroke Cerebrovasc Dis. (2012) 21:61–7. 10.1016/j.jstrokecerebrovasdis.2010.05.002 22225864 PMC3255072

[26] Ji R Shen H Pan Y Wang P Liu G Wang Y. Novel risk score to predict pneumonia after acute ischemic stroke. Stroke. (2013) 44:1303–9. 10.1161/STROKEAHA.111.000598 23482598

[27] Cugy E Sibon I. Stroke-associated pneumonia risk score: validity in a French stroke unit. J Stroke Cerebrovasc Dis. (2017) 26:225–29. 10.1016/j.jstrokecerebrovasdis.2016.09.015 27839768

[28] Harms H Grittner U Droge H Meisel A. Predicting post-stroke pneumonia: the PANTHERIS score. Acta Neurol Scand. (2013) 128:178–84. 10.1111/ane.12095 23461541

[29] Hassan AE Chaudhry SA Zacharatos H Khatri R Akbar U Suri MF. Increased rate of aspiration pneumonia and poor discharge outcome among acute ischemic stroke patients following intubation for endovascular treatment. Neurocrit Care. (2012) 16:246–50. 10.1007/s12028-011-9638-0 21993605

[30] Buchholz DW Bosma JF Donner MW. Adaptation, compensation, and decompensation of the pharyngeal swallow. Gastrointest Radiol. (1985) 10:235–9. 10.1007/BF01893106 4029539

[31] Hamidon BB Nabil I Raymond AA. Risk factors and outcome of dysphagia after an acute ischaemic stroke. Med J Malaysia. (2006) 61:553–7. 17623955

[32] Nowak K Derbisz J Peksa J Lasocha B Brzegowy P Slowik J. Post-stroke infection in acute ischemic stroke patients treated with mechanical thrombectomy does not affect long-term outcome. Postepy Kardiol Interwencyjnej. (2020) 16:452–9. 10.5114/aic.2020.101771 33598019 PMC7863840

[33] Vermeij JD Westendorp WF Dippel DW van de Beek D Nederkoorn PJ. Antibiotic therapy for preventing infections in people with acute stroke. Cochrane Database Syst Rev. (2018) 1:CD008530. 10.1002/14651858.CD008530.pub3 29355906 PMC6491314

[34] Vermeij JD Westendorp WF van de Beek D Nederkoorn PJ. Post-stroke infections and preventive antibiotics in stroke: Update of clinical evidence. Int J Stroke. (2018) 13:913–20. 10.1177/1747493018798557 30175940

[35] Vermeij JD Westendorp WF Roos YB Brouwer MC van de Beek D Nederkoorn PJ. Preventive ceftriaxone in patients with stroke treated with intravenous thrombolysis: post hoc analysis of the preventive antibiotics in stroke study. Cerebrovasc Dis. (2016) 42:361–9. 10.1159/000446160 27336314 PMC5296924

[36] Burkhardt JK Tanweer O Litao M Sharma P Raz E Shapiro M. Infection risk in endovascular neurointerventions: a comparative analysis of 549 cases with and without prophylactic antibiotic use. J Neurosurg. (2019) 132:797–801. 10.3171/2018.10.JNS182540 30738405

[37] Warnecke T Labeit B Schroeder J Reckels A Ahring S Lapa S. Neurogenic dysphagia: systematic review and proposal of a classification system. Neurology. (2021) 96:e876–89. 10.1212/WNL.0000000000011350 33318164

[38] Chumbler NR Williams LS Wells CK Lo AC Nadeau S Peixoto AJ. Derivation and validation of a clinical system for predicting pneumonia in acute stroke. Neuroepidemiology. (2010) 34:193–9. 10.1159/000289350 20197702 PMC2883837

