# Supplementary material for: Predicting Poststroke Pneumonia in Patients With Anterior Large Vessel Occlusion: A Prospective, Population-Based Stroke Registry Analysis
Source: Front Neurol. 2022 Feb 17;13:824450. doi: 10.3389/fneur.2022.824450 (PMC8893016; doi:10.3389/fneur.2022.824450)
Supplement: Supplementary file 1 [file Table_1.pdf]

**Supplement table 1: comparison of large vessel occlusion patients with and without endovascular therapy (EVT)**				
		EVT (n = 2132)	no EVT (n = 2149)	p-value[1]
sociodemographic factors				
sex, % (n)	female	53.4 (1139)	56.1 (1206)	**n. s.**
	male	46.6 (993)	43.9 (943)	**n. s.**
age, years	mean (±SD)	73.44 (±13.3)	76.41 (±13.1)	**< 0.001**
age categories, % (n)	< 65	23.2 (494)	17.7 (380)	**< 0.001**
	≥ 65	76.8 (1638)	82.3 (1769)	**< 0.001**
	≥ 75	56.1 (1197)	63.1 (1355)	**< 0.001**
mRS at admission	median (IQR)	5.0 (4.0 – 5.0)	4.0 (3.0 – 5.0)	**< 0.001**
	≥ 4	84.8 (1807)	66.9 (1437)	**< 0.001**
time onset to admission, % (n)	< 4 hours	59.2 (1356)	40.8 (936)	**< 0.001**
care before admission, % (n)	independent at home	84.8 (1807)	73.1 (1571)	**< 0.001**
	care at home	9.0 (191)	13.3 (286)	**< 0.001**
	care in institution	6.3 (134)	13.6 (292)	**< 0.001**
duration of hospital stay, days	mean (±SD)	12.6 (±10.26)	9.8 (±8.3)	**< 0.001**
prior anticoagulants, % (n)	none	80.1 (1708)	82.5 (1772)	**n. s.**
	vitamin K antagonist	7.3 (155)	5.7 (123)	**n. s.**
	NOAC	12.6 (269)	11.8 (254)	**n. s.**
symptoms at admission				
initial NIHSS, % (n)	median (IQR)	15 (10 – 18)	11 (5 – 17)	**< 0.001**
	≤ 4	6.4 137	22.8 489	**< 0.001**
	5 - 15	49.0 1044	43.5 935	**< 0.001**
	16 -20	30.2 643	21.6 465	**< 0.001**
	≥ 21	14.4 308	12.0 258	**< 0.05**
aphasia, % (n)		59.6 (1270)	55.4 (1191)	**< 0.05**
dysphagia, % (n)		59.1 (1259)	47.9 (1030)	**< 0.001**
dysarthria, % (n)		59.8 (1274)	55.1 (1184)	**< 0.01**
motor impairment, % (n)		93.5 (1994)	82.2 (1766)	**< 0.001**
impaired consciousness, % (n)	(sopor or coma)	26.2 (558)	25.1 (539)	**n. s.**
large vessel occlusion (isolated)		% n	% n	
ICA, carotid triangle		19.1 408	28.5 613	**< 0.001**
MCA, M1 segment		61.6 1314	36.1 775	**< 0.001**
MCA, M2 segment		19.2 410	35.4 761	**< 0.001**
comorbidities		% n	% n	
atrial fibrillation		47.8 1013	42.8 861	**< 0.01**
hypertension		82.9 1756	84.4 1699	**n. s.**
diabetes mellitus		21.1 447	24.3 489	**< 0.05**
treatment		% n	% n	
i.v. thrombolysis		56.5 1204	33.1 711	**< 0.001**
admission intensive care unit		65.7 1392	18.3 369	**< 0.001**
poststroke pneumonia		24.9 530	14.1 302	**< 0.001**

EVT = endovascular treatment; mRS = modified Rankin Scale; NIHSS = National Institute of Health Stroke Scale; ACI = internal carotid artery, IQR = interquartile range; MCA = middle cerebral artery; [1] calculated between both EVT categories using 2-sided Chi^2 – Test or Mann-Whitney-U-Test, as applicable. n. s. = not significant.
